# Prospects of Cytomegalovirus-Specific T-Cell Receptors in Clinical Diagnosis and Therapy

**DOI:** 10.3390/v15061334

**Published:** 2023-06-07

**Authors:** Xuejie Li, Hanying Liang, Jun Fan

**Affiliations:** State Key Laboratory for Diagnosis and Treatment of Infectious Diseases, The First Affiliated Hospital, College of Medicine, Zhejiang University, Hangzhou 310003, China

**Keywords:** HCMV, diagnosis, treatment, adoptive immunotherapy, immune response, TCR

## Abstract

Human cytomegalovirus (HCMV) is responsible for widespread infections worldwide. In immunocompetent individuals it is typically latent, while infection or reactivation in immunocompromised individuals can result in severe clinical symptoms or even death. Although there has been significant progress in the treatment and diagnosis of HCMV infection in recent years, numerous shortcomings and developmental limitations persist. There is an urgent need to develop innovative, safe, and effective treatments, as well as to explore early and timely diagnostic strategies for HCMV infection. Cell-mediated immune responses are the primary factor controlling HCMV infection and replication, but the protective role of humoral immune responses remains controversial. T-cells, key effector cells of the cellular immune system, are critical for clearing and preventing HCMV infection. The T-cell receptor (TCR) lies at the heart of T-cell immune responses, and its diversity enables the immune system to differentiate between self and non-self. Given the significant influence of cellular immunity on human health and the indispensable role of the TCR in T-cell immune responses, we posit that the impact of TCR on the development of novel diagnostic and prognostic methods, as well as on patient monitoring and management of clinical HCMV infection, will be far-reaching and profound. High-throughput and single-cell sequencing technologies have facilitated unprecedented quantitative detection of TCR diversity. With these current sequencing technologies, researchers have already obtained a vast number of TCR sequences. It is plausible that in the near future studies on TCR repertoires will be instrumental in assessing vaccine efficacy, immunotherapeutic strategies, and the early diagnosis of HCMV infection.

## 1. Introduction

Human cytomegalovirus (HCMV), a member of the beta subfamily of the Herpesviridae family, also known as human herpesvirus 5, is the largest of the human herpesviruses. As an opportunistic pathogen, HCMV is prevalent worldwide, with infection rates approaching 100% in developing countries [1]. After primary infection, it establishes lifelong latency [2]. While primary HCMV infection is typically asymptomatic in immunocompetent individuals [3], a minority might present with clinical symptoms following infection [4]. The factors influencing this variable clinical presentation are yet unknown. Nonetheless, viral infection or reactivation can occur in immunocompromised individuals, such as those with T-cell deficiency or dysfunction following hematopoietic stem cell transplantation (HCT). This often leads to life-threatening multi-organ diseases and significant morbidity and mortality [5].

HCMV, the largest DNA virus among human herpesviruses [6], possesses a genome length of 240 kb. It comprises the unique long component sequence (UL) and the unique short component sequence (US), which encompass 208 open reading frames and encode up to 227 proteins. These proteins, categorized as immediate early (IE), early (E) and late (L) proteins according to their properties, are products of HCMV genome expression at different stages. Individually or in combination, these antigens or proteins effectively induce specific cellular immunity [7]. T-cell immune responses to HCMV are largely specific [8]. A vast array of antigens expressed at various stages of viral replication engage in activating HCMV-specific CD8 + and CD4 + T-cells [3]. The most commonly targeted HCMV epitopes for immune activation include IE-1, pp65, and UL148 proteins [3,8]. However, opinions differ regarding the most dominant immune antigens and the selection of antigenic peptides.

Present strategies to prevent and treat HCMV encompass antiviral drugs, vaccines, and adoptive immunotherapy. However, the effectiveness of antivirals is often limited by their toxicity and cross-resistance. Fortunately, numerous studies suggest immunotherapy as a promising approach to counteract the side effects of antiviral therapy and to develop preventive measures. Novel vaccine therapies and immunotherapies have been tested in several clinical settings. While these therapies have not yet been widely integrated into standard practice, they demonstrate promising applications. Furthermore, existing routine laboratory tests cannot accurately stratify the risk for HCMV infection, nor can these tests guide targeted preventive and therapeutic strategies. The lack of effective prevention, treatment, and timely diagnosis of HCMV may be attributable to the fact that the immune mechanism of HCMV remains unclear. It is hypothesized that T-cellular immune reconstitution is vital for long-term control of HCMV reactivation [9], and T-cells play a significant role in cellular immune responses against HCMV infection. Recent studies suggested that variations in the effectiveness of antiviral therapy between patients might be closely associated with the immune responses of HCMV-specific T-cells. The TCR, which is central to T-cell immune responses, has become a focus of interest in numerous disease areas. Consequently, TCR-based immune-specific therapies have been continuously developed to control HCMV. Moreover, an analysis of TCR repertoires can provide insights into an individual’s history of antigen exposure and can help in exploring the etiological mechanisms of immune-related diseases. Studying specific T-cells and their TCR repertoire could aid in the identification of potential diagnostic biomarkers and the development of safe and effective immunotherapies. This, in turn, offers a reliable foundation for the clinical application of adoptive immunotherapy with HCMV-specific T-cells and TCR T-cells.

## 2. Immune Response to CMV Infection

**Figure 1:**
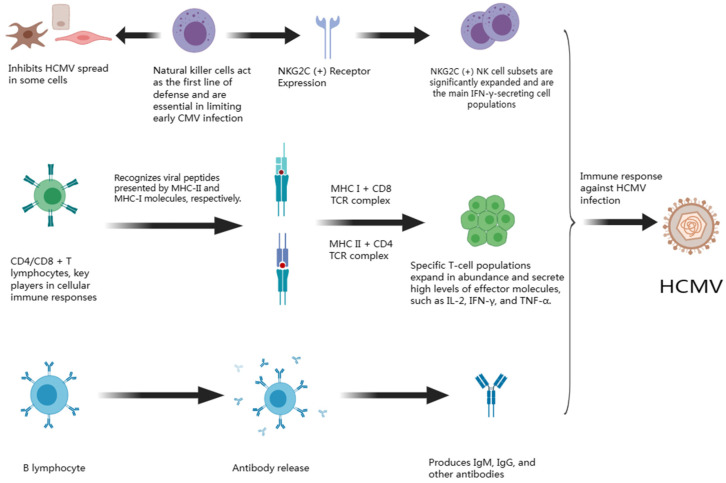
HCMV infection-associated immune response. Figures in this review are drawn using MedPeer (www.medpeer.cn).

The severity of HCMV infection is directly influenced by the level of immune response. Cell-mediated immunity is vital in combatting HCMV infection. In vivo, HCMV infects a wide variety of cells, thereby providing conditions for its latency and proliferation. It is currently thought that αβ T-cells, γδ T-cells, natural killer (NK) cells, and B cells play roles in inhibiting HCMV infection [10,11]. The pathophysiological process of HCMV invasion in humans triggers a complex cascade of immune responses, involving multiple signal transduction pathways throughout the peripheral immune system. This reaction is not restricted to individual immune cells or specific immune molecules. For instance, T-cell antigen recognition and differentiation can be impaired, natural killer (NK) cell killing can be inhibited, and the secretion of proinflammatory cytokines can be reduced (Figure 1).

### 2.1. Humoral Immune Response

B cells, which produce antibodies, occupy a unique position in the immune system. In addition to the cellular immune responses that play a pivotal role, humoral immune responses also contribute to adaptive immunity in response to HCMV infection. However, the relative importance of humoral immunity in humans remains uncertain as no direct evidence has been provided to date. While some studies have suggested that high antibody titers can improve outcomes following HCMV infection in hematopoietic stem cell transplantation (HSCT) recipients, other investigations have refuted this conclusion. Clinically, humoral immunity has, at best, demonstrated a role in managing HCMV infection. Immune responses mediated by B-cells have shown only partial protection against HCMV infection. Traditionally, protection against HCMV has been deemed to be more dependent on T-cells as animal experiments have revealed that protection can be entirely mediated by T-cells in scenarios of B cell depletion [12]. Individuals with immunodeficiency who are incapable of producing specific antibodies do not exhibit an increased risk for HCMV disease, suggesting that antibodies are not essential for HCMV resistance. HCMV IgG levels are inversely correlated with T-cell receptor diversity [13]. Moreover, a positive relationship has been found between HCMV IgG antibody levels and the count of HCMV-specific T-cells in certain transplant recipients [14]. It appears that HCMV antibodies might bolster cellular immunity. Although recent studies have begun to shed light on the relationship between HCMV humoral and cellular immunity, more concrete results are necessary. A protective effect of neutralizing antibodies against HCMV infection has been shown in various experimental and clinical studies, although such antibodies do not prevent HCMV infection caused by direct cell-to-cell transmission [15,16,17]. The current controversy revolves around choosing the best antigenic target among the abundant HCMV surface proteins. The antigenic targets that can stimulate potent monoclonal neutralizing antibody responses across various target cells remain elusive, which has hindered the development of an HCMV-neutralizing antibody vaccine. Furthermore, despite the significance of neutralizing antibodies, their detection methods are complex and standards are inconsistent, something which currently limits their clinical utility. Several other challenges persist in the realm of HCMV neutralizing antibody research and vaccine development.

### 2.2. Innate Killer Cell Immune Response

The innate immune system plays a vital role in defending against HCMV infection and triggering adaptive immune responses. HCMV primarily stimulates innate immune cell surface receptors within the body, activating intracellular signal transduction pathways. This leads to the production of various cytokines, including class I interferons and inflammatory cytokines, further recruiting other innate immune cells such as natural killer (NK) cells, and providing activation signals for adaptive immune responses to initiate T- and B-cell-specific immune responses [18,19]. Historically, NK cells have been categorized as innate immune cells. However, recent discoveries have revealed that they possess features of adaptive immunity, including antigen-driven clonal expansion and long-term memory formation, and play a significant role in antiviral and antitumor responses [20]. NK cells have been shown to play a crucial role in haploidentical hematopoietic stem cell transplantation. With the global outbreak of novel coronaviruses in 2019, the importance of NK cells in controlling various viral infections has gained researchers’ attention. Numerous studies in mice and humans have demonstrated NK cells’ integral role in the innate immune system against HCMV infection. The study of NK cell immune responses to MCMV in mouse models has provided valuable insights into the interaction between NK cells and HCMV. The activation of resting NK cells is tightly controlled by the balance of inhibitory and activating surface receptors, a characteristic distinguishing them from T and B cell receptors which require genetic rearrangements. As the first lymphocyte population to recover after HSCT, NK cells play a pivotal role in protecting recipients from tumor relapse and viral infection prior to the full recovery of T-cell immunity. Studies have noted an increased susceptibility to viruses in patients with NK cell deficiencies [21]. NK cells can trigger immune responses to HCMV by directly recognizing HCMV virions [22]. Infection with or reactivation of HCMV induces swift maturation of NK cells and may profoundly impact the NK receptor repertoire. In some healthy individuals, HCMV infection can alter the phenotypic and functional characteristics of NK cells. In animal models, NK cells develop specific immune responses when stimulated with MCMV antigens. About half of the NK cells in mice express the Ly49H receptor, which specifically recognizes viral proteins on the surface of MCMV-infected cells. MCMV replicates at high levels in mice lacking the Ly49H receptor [23], while resistance to MCMV infection significantly increases in mice with transgenic expression of the Ly49H receptor [24]. Adaptive NK cell production has also been observed in human studies. In one study, an increased frequency of NKG2C (+) NK cells was observed in CMV-seropositive healthy individuals compared to seronegative populations. Expression of the NKG2C receptor on the surface of human adaptive NK cells was increased, which mirrored the increase in Ly49H receptor expression on the surface of mouse NK cells induced by MCMV, and the NKG2C receptor could recognize CMV antigen and expand. NK cells returned to normal numbers several weeks after transplantation, and the killing function returned to normal within 1 year [25]. In another study, HCMV infection or reactivation after HSCT significantly accelerated NK cell reconstitution, with substantial expansion of NKG2C (+) NK cell subsets, which were the primary IFN-γ-secreting cells [26]. NK cell phenotype and function can serve as biomarkers to predict HCMV risk [27]. However, some studies have disputed the effectiveness of NKG2C (+) NK cells against HCMV infection or reactivation after transplantation [28]. Further research is required to elucidate the causal relationship between the expansion of adaptive NK cells and HCMV infection. In addition, the conditions necessary for the reconstitution of adaptive NK cells demand further investigation.

### 2.3. T-Cell Immune Response

T-cells are critical effector cells of the adaptive immune system. In 1977, it was discovered that [29] immune mechanisms linked with T lymphocytes appeared to be crucial for recovery from murine cytomegalovirus (MCMV) infection. While other viruses triggered the mobilization of no more than 1% of T-cells, up to 50% of T-cells responded during a CMV infection. T-cells directly targeted numerous HCMV peptides [30] and stringently regulated HCMV latency and reactivation [31].

T-cells primarily include CD4 + T-cells and CD8 + T-cells, which produce immune responses in vivo by recognizing exogenous and endogenous antigenic peptides bound to and presented by the major histocompatibility complex (MHC) II and MHC I molecules, respectively. T-cells target HCMV-infected cells via cytotoxicity, which subsequently induces T-cell clone proliferation, differentiation, memory establishment, and recruitment of virus-specific subsets [32]. Circulating T-cell populations and T-cell phenotypes in healthy individuals are profoundly impacted by HCMV infection [33]. HCMV immune responses are characterized by a massive expansion of T-cell populations [34], with HCMV-specific CD8 + T-cells being particularly prevalent [35]. CD8 + T-cells were the earliest and most researched factor associated with HCMV protection. Numerous experiments indicated [36] that virus-specific CD8 + T-cells play a vital role in preventing HCMV disease. The quantity of CD8 + T lymphocytes found in hematopoietic stem cell transplant recipients predicts the risk for HCMV infection in these recipients and has been linked with HCMV activation, associated disease protection, and immune recovery [37]. In heart, lung, liver, and kidney transplantation, the number of HCMV-specific CD8 + T-cells helps determine the recipient’s risk of developing active HCMV infection. This measurement is associated with HCMV-related disease and the maintenance of allograft function, and has proved to be of great value in post-transplantation recipient management [38,39]. The median number of CD8 + T-cells in the peripheral blood of healthy virus carriers is 10%, while in older adults this figure rises to 40% specifically for HCMV antigens [3]. In immunocompetent mice and humans, latent HCMV infection contributes to “memory inflation” of memory CD8 + T-cells, a process possibly due to repeated exposure to CMV antigens, resulting in long-term expansion of HCMV-reactive memory T-cells [40,41]. Several aspects remain unclear in the study of HCMV-specific CD8 + T lymphocytes, such as the selection of specific antigens, the selection of antigen peptides, and the significance of HCMV-specific T-cell number and function. CD4 + T lymphocytes generally make up a large portion of T-cells in healthy people. The traditional view posits that CD4 + T-cells exert immune effects through helper CD8 + T-cells. CD4 + T-cells primarily play a role in antiviral immunity in two ways: first, they recruit CD8 + T lymphocytes and B cells to the site of infection; second, they regulate the expansion and function of other effector cells via cytokines [42]. Recently, CD4 + T-cells have also been recognized to play an important role in the control of viral infections [43]. It has been shown previously that [44] the persistence of transferred cytotoxic CD8 + T-cells can be facilitated by the restoration of CD4 + human cytomegalovirus-specific helper T-cell responses. Progressive depletion of CD4 + T-cells increases susceptibility to various pathogens, including herpes viruses [45]. Persistent dissemination of HCMV has also been associated with a deficiency in CD4 + T-cells [46]. Some late-differentiated D4 + T-cells, specifically for HCMV, have been observed to expand abnormally in infected individuals. These cells produce granules and perforin with direct cytolytic capacity [47]. During the acute phase of the response to HCMV infection, specific CD4 + T-cells are highly activated and proliferate. In vitro experiments and observational studies across different populations have also emphasized the importance of CD4 + T lymphocytes in the HCMV immune response. In pregnant women with primary CMV infection, the presence of HCMV-specific memory CD4 + T-cells has been linked with rapid control of HCMV infection and a lower risk for vertical transmission of the virus to the fetus [48]. The count of HCMV-specific CD4 + T-cells has been found to correlate with viral load, complications, and post-transplant prognosis in various organ transplant recipients [49]. Notably, CD4 + T-cells have been found to be essential for controlling HCMV infection [50]. However, most current studies on the protective mechanism of CD4 + T-cells have focused on cytokines and surface molecules, with few studies revealing the underlying response mechanism. Elucidation of the development of cellular immune responses can foster improved vaccine development and adoptive immunotherapy.

## 3. T-Cell Receptor

The T-cell repertoire covers a vast array of antigens and includes highly polymorphic TCRs [51]. These TCRs from the cornerstone of T-cell immune responses. Each individual’s T-cells contain a repertoire of millions of unique receptors [52].

The TCR is a heterodimeric transmembrane protein composed of either α/β or γ/δ chains [53,54]. In peripheral blood, αβ T-cells constitute more than 90% of the T-cell population. There are several differences between γδ T-cells and αβ T-cells, such as variation in antigen recognition, activation, antigen-specific repertoire formation, and effector function [55]. While most αβ TCRs require binding to peptide fragments MHC, γδ TCRs, in contrast, do not need MHC-mediated antigen presentation and no universal requirement for coreceptor interaction has been identified. The exact mechanism of γδ T-cell function remains elusive. TCRs recognize specific antigens within the context of peptide MHC complexes [56]. TCR clonotypes shared between individuals play a pivotal role in pathogen-specific immune responses and infection control [57]. Different individuals often [58,59] use the same or similar TCRs for at least one of the chains of a heterodimer in antigen recognition. This sharing of TCRs is particularly pronounced in chronic or latent viral infections, such as HCMV [60]. TCR α and β chain sites include variable (V) and joining (J) gene segments. Moreover, the β-chain includes a diversity (D) gene segment which is absent in the α-chain. The expression of TCR-specific sequences is generated by rearrangements, insertions, mutations, and deletions of V, D, and J gene segments. These rearrangements create a diverse TCR repertoire capable of recognizing a broad range of antigens, thereby providing effective antiviral immunity [61]. Almost no two identical V (D) J somatic rearrangements occur within any individual [62]. The V region of the α and β chains has three complementarity-determining regions (CDRs), with CDR3 being the most variable [63]. This region, which directly contacts the antigen, largely determines the binding specificity of the TCR to the antigen MHC complex. Upon stimulation with various antigens, TCR V region genes generate specific recognition, enabling dominant expansion of T-cells carrying such genes. Specific CDR3 amino acid sequences are abundantly expressed, and their lengths show significant variation. Previous studies have found that virus-specific TCRβ clonotypes have longer TCRβ CDR3 lengths compared to autoantigen-specific clonotypes [64]. CDR3 sequence analysis allows for the tracking and quantification of T-cell clones within different tissues, time points, individuals, and even species [65,66]. The analysis of the total CDR3 length and its distribution has been used to measure the degree of clonality and diversity of T-cells during immune responses.

**Figure 2:**
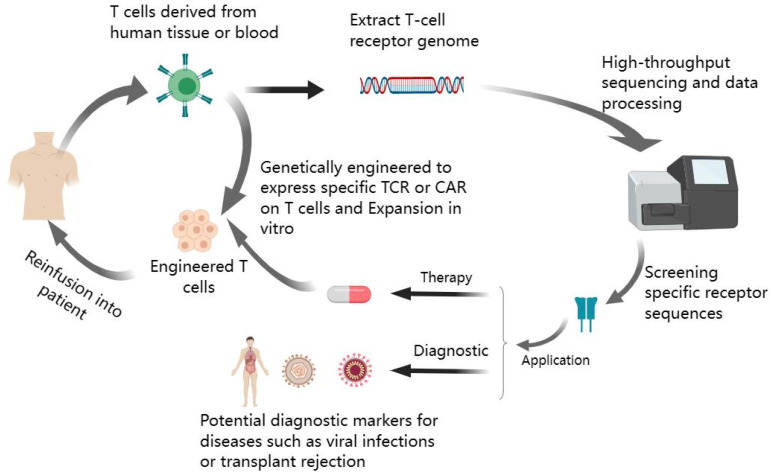
Specific T-cell receptor sequences in diagnosis and therapy.

Several studies have demonstrated a connection between TCR repertoire diversity and immunity, with specific receptor sequences playing a role in predicting disease risk [67,68,69]. The particular characteristics of the TCR used are key determinants of the success of TCR gene therapy [70]. Moreover, TCR repertoire information enables the customization of immunization strategies for neoantigens to align with the patient’s T-cell repertoire [71]. Most current sequencing analyses of TCRs have focused on the β-chain. Given that each αβ T-cell expresses only one variant of the β-chain, different β-chain numbers could reflect the number of clonotypes. The T-cell repertoire can be assessed by sequencing the β chain of the TCR. The α-chain, which lacks the D gene segment and typically does not contact the antigen, carries less information [72]. However, α-chain sequencing can confirm clonality and provide more detailed insight about the cell repertoire. Combining total mRNA and VDJ sequencing could help clarify the transcriptional heterogeneity of both antigen-specific and non-specific lymphocytes, and the clonal relationships among different T-cell subsets. TCR sequencing has been a topic of interest in many areas of disease study [73,74,75]. Its sequence can serve as a unique identifier for T-cell clones, enabling the monitoring of clonal dynamics and heterogeneity of T-cell responses, detecting immune status, and assisting with diagnosis. For instance, Hou et al. [76] discovered a set of TCR sequences related to SARS-CoV-2 that could serve as potential diagnostic markers for COVID-19 patients. Other researchers [77] have analyzed the TCRα repertoire to develop biomarkers that aid the diagnose of primary immunodeficiency. In addition, prognostic risk models based on TCRs have been explored to effectively predict the prognosis of breast cancer patients [73]. One study sequenced the TCR repertoire and identified three sequences that specifically bind HCMV [78]. Specific antigen recognition can bias the TCR repertoire toward antigen-specific T-cells [79]. Dominant T-cell clones have potential as early diagnostic biomarkers for T-cell-mediated rejection in renal transplantation [80]. (Figure 2)

HCMV infection triggers highly complex immune responses, resulting in HCMV-specific TCRs that are also exceptionally diverse and variable. Related studies have shown a strong correlation between the diversity of HCMV-specific TCR sequences and the activation state of HCMV [81,82]. HCMV-specific T-cell clones influence the clonotypes within the repertoire, and they also promote the expansion of a limited number of non-HCMV T-cell populations [83]. Wang et al. [13] revealed that the diversity of CD8 + T-cell clonotypes plays a significant role in controlling adverse effects caused by CMV infection. In their study, HCMV-specific public TCRα and β sequences were heavily used. Human leukocyte antigen (HLA)-A24-restricted HCMVPP65-specific cytotoxic T-cells have longer amino acid sequences in the TCR-CDR3 region than do HLA-A2 CTLs [81]. The BV and BJ genes involved vary depending on the target antigen and HLA. For example, BV7 and BV12 reportedly dominate HLA-A02-restricted HCMV PP65-specific cytotoxic T-cells with BJ14 and BJ12 [84]. Table 1 lists some reported HLA-A02-restricted HCMV-associated TCR sequences (Table 1). High-throughput technologies have generated growing amounts of HCMV-associated TCR sequencing data, as well as epitope and immune receptor databases. These data have demonstrated that our previous understanding of the mechanisms of the adaptive immune system in controlling HCMV was far from complete. High-throughput sequencing technologies now make it possible to analyze millions of T-cells in a single experiment [62]. Single-cell sequencing technology can effectively address the issue of cell population heterogeneity to obtain comprehensive receptor information of individual cells [85,86]. The advancement of sequencing technology has greatly facilitated the study of HCMV-specific TCR sequences, the understanding of the immune reconstitution process after viral activation, and the exploration of the mechanism underlying the immune response between T-cells and HCMV (Table 1).

## 4. Diagnosis of HCMV

Early and prompt diagnosis of active HCMV infection is crucial to mitigate or even prevent severe clinical consequences resulting from viral activation. It can also support early clinical screening, timely treatment, and accurate prognosis. Several laboratory tests are available for this, including traditional viral isolation and culture, antigen-antibody testing, viral genetic testing, and immune monitoring. While histopathology remains the gold standard [91], the complex process of virus isolation and culture is not conducive to early diagnosis of HCMV infection, and is primarily used for scientific research purposes. Faster and more sensitive detection methods are gradually replacing virus isolation and culture, enabling real-time quantitative detection and monitoring of the virus. The PP65 antigen, encoded by UL83 in the HCMV genome, is a major component of the viral capsule. During latent HCMV infection, the expression of PP65 is minimal, which makes it a commonly used indicator for diagnosing active HCMV infection. The PP65 antigenemia test, which detects the presence of PP65 antigen in peripheral blood leukocytes through specific antibody-antigen binding, is a semiquantitative method. However, due to the lifespan of neutrophils, this test requires prompt processing of clinical specimens within hours. In addition, its results may be influenced by different assay probes or the antibodies used, as well as neutrophil counts from whole blood samples. However, it is faster and also more cost-effective than quantitative fluorescence polymerase chain reaction (qPCR). In addition, the results exhibit better correlations with clinical symptoms in immunocompromised patients [92]. HCMV antibody detection methods are categorized into three main groups based on the antigens used: those based on viral lysate antigens, genetically engineered recombinant protein antigens, and chemically synthesized polypeptide antigens. HCMV IgM and IgG antibodies, along with antibody affinity, are commonly used to determine the status of HCMV infection in the body. HCMV IgM and IgG antibodies indicate primary and previous HCMV infections, respectively [93]. The affinity indicator of HCMV IgG antibodies reflects the activity of HCMV infection and provides some complementary diagnostic value for HCMV infection [94]. Genetic monitoring has emerged as a preferred method to quickly determine whether CMV is replicating in a patient. Timely monitoring of genetic changes related to HCMV is helpful in rapidly diagnosing HCMV infection and disease, guiding antiviral therapy, and monitoring treatment response [95]. HCMV viral load serves as guide for preventive therapy to control HCMV infection, while HCMV RNA is a highly specific indicator of CMV replication which no commercial assays currently available are able to detect. Consequently, qPCR for HCMV DNA in blood has become the preferred diagnostic test due to its high sensitivity and throughput. Determining the rate of increase in viral load has been used to assess the risk of developing HCMV disease [96]. Nevertheless, challenges related to variability between PCR detection platforms and assays persist [97].

Immunological monitoring of nonspecific and HCMV-specific T-cell numbers and function has emerged as a valuable clinical tool for HCMV risk stratification and management following solid organ transplantation [98]. The detection of HCMV-specific cell-mediated immunity can be useful to determine [91,99] viral load and optimize the use of antiviral drugs in preemptive treatment strategies. Furthermore, specific cell-mediated immunity tests can be used alone or in combination with serology tests to assess risk prior to transplantation. Such methods primarily utilize overlapping peptide libraries to stimulate CD4 + and CD8 + T-cells [99]. Subsequently, the production of cytokines (such as IFN-γ) or cell proliferation is measured using flow cytometry or enzyme-linked immunosorbent assay (ELISA), with the former generally offering higher sensitivity [100]. However, that method is challenging to standardize and can only analyze intracellular events, failing to detect biologically active cytokines secreted during the stimulation period. ELISA-based assays require small blood volumes and are straightforward to perform [101]. However, they are limited to detecting IFN-γ-producing CD8 + T-cells and do not allow for detection at the single-cell level. On the other hand, the enzyme-linked immunospot assay (ELISPOT) is particularly useful for detecting low-level responses and can measure biologically active cytokine-secreting cells at the single-cell level [102]. ELISPOT exhibits greater sensitivity than intracellular cytokine staining for detecting antigen-specific cells. Nonetheless, that method lacks functional readouts, is restricted to certain HLA types, and is not suitable for routine diagnostic use.

## 5. Treatment of HCMV

Current treatment options focus on inhibiting the replication process of the HCMV virus [103]. The introduction of prophylactic strategies has led to a shift in the epidemiology of HCMV towards late-onset disease. Ganciclovir has been widely used as the primary antiviral agent for prophylactic treatment of HCMV. Preemptive administration of ganciclovir to asymptomatic patients with HCMV DNA or antigenemia has been found to significantly reduce the risk of HCMV disease [104]. Letermovir, a novel antiviral agent, has shown good clinical efficacy in preventing HCMV. However, it may be associated with an increased incidence of advanced HCMV and may delay the immune reconstitution of HCMV-specific T-cells. While antiviral drugs are recommended as first-line therapy for HCMV, they can be toxic and are susceptible to drug resistance. In addition, their effectiveness is limited in the absence of cell-mediated immune responses. HCMV remains a significant cause of morbidity and mortality following allogeneic hematopoietic stem cell transplantation, despite the use of preemptive treatments and effective prophylactic antiviral drugs [105,106]. Antibody products and vaccines have shown promise for the prevention and treatment of HCMV. HCMV immunoglobulin not only prevents HCMV entry into host cells, but also enhances cytotoxicity and complement-mediated cytolysis. Although the use of immunoglobulin in combination with antiviral agents is recommended for the treatment of HCMV, conclusive evidence is still lacking. In recipients of hematopoietic stem cell transplantation (HSCT), prevention or treatment of HCMV with immunoglobulins is often ineffective [107].

The development of HCMV vaccines has been a subject of research for decades, primarily focused on preventing or mitigating congenital HCMV infection. However, to date, most vaccines have failed to demonstrate clinical safety and efficacy, and no vaccine has been licensed. In theory, vaccines could directly target HCMV reactivation and host defects in disease, including protective immune responses, to control viral reactivation and prevent disease. Several vaccines [108,109] have shown successful induction of potent and broad T-cell responses. Vaccine efficacy may also depend on TCR clonality within polyclonal antigen-specific T-cell populations [110]. Therefore, the importance of antigen-specific T-cell repertoire diversity should be considered when designing prophylactic T-cell vaccines. There are several major challenges in the development of HCMV vaccines, including the selection of appropriate targets and optimal vaccination schedules, and the choice of suitable endpoints and populations for clinical trials. Although prophylactic vaccines offer an ideal approach to treating and preventing HCMV, the complex interactions between the adaptive and innate immune systems and HCMV itself present significant challenges in vaccine research.

The emergence of T-cell-based immunotherapy has revolutionized our understanding of the role of T-cells in combating various diseases, including viral infections, autoimmune disorders, and malignancies. The clinical use of adoptive HCMV-specific T-cell therapy against viral infections was first implemented in 1992 [111]. Infusion of virus-specific T-cells has been explored as a strategy to treat or prevent HCMV disease by directly restoring CMV-specific cellular immunity. In recent years, adoptive immunotherapy using HCMV-specific cytotoxic T-cells (CTLs) has emerged as a promising method [112]. Optimal costimulation of virus-specific T-cells through binding to their native TCRs has been shown to promote long-term persistence of these cells in patients [113,114]. Experimental studies have demonstrated that the adoptive transfer of HCMV-specific T-cells facilitates the reconstitution of functional and durable virus-specific T-cell repertoires. This strategy can significantly reduce or even prevent HCMV disease, progressive viral infections, and persistent complications, thereby sparing [115,116] patients from prolonged antiviral therapy. However, a drawback of this approach is that the expansion of CTLs is time-consuming, and the efficiency of expansion can vary widely among individuals, which may result in some patients missing their therapeutic window when in urgent need of treatment.

Studies have indicated that TCR T-cell therapy holds greater potential as HCMV therapy than virus-specific cytotoxic T-cell therapy [117]. Adoptive cell transfer therapy (ACT) has emerged as a highly promising approach in cancer immunotherapy. Two notable forms of ACT are TCR-engineered T-cell therapy (TCR-T) and CAR T-cell therapy, which confer antigen specificity through genetic modification of T lymphocytes [118]. CAR-T therapy, including CAR-T targeting HCMV, enables direct antigen recognition independent of MHC restriction [119]. It allows for the development of autologous virus-specific CTLs without relying on HLA restriction [120]. However, it requires a complex process, leading to prolonged manufacturing time, high costs, and challenges in scaling up the process [121,122]. Moreover, it can lead to toxicities such as cytokine release syndrome. A recent innovative study [123] utilized CRISPR-Cas9 technology to generate non-viral and gene-specific CAR-T-cells, eliminating the need for viral vectors. This approach simplifies manufacturing, reduces preparation time and costs, and enhances the safety and efficacy of CAR-T products. In contrast with CAR-T, TCR-T therapies are based on native T-cells and their endogenous TCRs, rather than antibody-based CARs [124]. The study of redirected T-cell specificity through TCR transfer, reported in 1986, ref. [125] has greatly advanced TCR-T therapy and its application. TCR-T adoptive immunotherapy has shown promise for the treatment of various diseases, including viral infections, offering new possibilities for therapeutic approaches [126]. TCRs recognize HLA-presented peptides derived from various cellular proteins [127]. TCR-based ACT has been found to be efficacious for certain hematological malignancies and viral-associated malignancies [128]. TCR-T exhibits high sensitivity in recognizing target antigens even at low concentrations [129]; it also offers cost advantages through TCR transduction [130]. Transgenic TCR-based ACTs enable precise redirection of T-cell specificity in response to viral infection. Although TCR-based ACTS hold promise, there are limitations and challenges to overcome. These include MHC restriction, the selection of TCRs with optimal affinity for recognizing MHC, the choice of appropriate target antigens in the context of specific HLA genes, and more. The specific proteins and epitopes targeted by the TCR directly influence the expected frequency, affinity, and potential escape mechanisms within the TCR repertoire [131]. Optimal efficacy of TCR-T treatment may be achieved through the selecting of uniformly expressed antigens to minimize the variability in the TCR repertoire induced by immunodominant HCMV antigens.

HLA dependency has emerged as a significant limitation of TCR-T therapy. Most current studies have focused on targeting a single HLA type, thereby excluding many patients with different HLA types from receiving TCR-T-cell treatment. Future research should expand the coverage of TCR-T-cell therapy to encompass a broader range of patients with diverse HLA types. Advancements in computer technology may aid the prediction of potential HCMV epitopes, facilitating the identification of candidate targets. Another challenge associated with TCR gene therapy is TCR mismatch. Mismatches between introduced TCR α and β chains and the endogenous β and α chains can result in the generation of novel TCR chains with unknown risks and potential complications. However, several strategies have been proposed to address this issue. These include introducing additional disulfide bonds between TCR chains or modifying the structure of TCR chains to mitigate the risks associated with TCR mismatch [132].

## 6. Conclusions

HCMV is a highly prevalent and complex pathogen that poses significant challenges in prevention, diagnosis, and treatment. While some progress has been made in recent years, several limitations persist. These include issues related to antiviral drug toxicity, drug resistance, and cost-effectiveness. Furthermore, there has been a lack of substantial progress in the development of targeted vaccines. Current diagnostic methods often fail to accurately stratify the infection risk, leading to overtreatment and associated complications. Although new treatments have improved the management of HCMV infection, the development of and research into HCMV vaccines and immunotherapy remain uncertain. Studying the intricate dynamics of T-cell responses to viruses holds promise for elucidating the conditions necessary for healthy immune responses and the persistence of functional antigen-specific cells. Such information would greatly contribute to the design of vaccines that induce optimal cellular immune responses and long-lasting T-cell memory. Analysis of the TCR repertoire offers opportunities to develop novel technologies for clinical diagnosis and to design T-cell-based therapies, laying the foundation for immunodiagnosis and cellular immunotherapy. The mechanisms of HCMV immunoprotection are complex, and there is a scarcity of reports on how specific TCR-T-cells function in immune-compromised patients with HCMV infection. The study of the TCR repertoire in terms of the diagnosis, clinical prognosis, and treatment of HCMV is still in its early stages. The development of next-generation sequencing and single-cell TCR sequencing technology has enabled researchers to gain deeper insights into HCMV-induced changes in the TCR repertoire, providing valuable information on the specific immune response mechanisms against HCMV and immune reconstitution. However, the interpretation of TCR sequences remains limited by the diversity of the TCR repertoire and scarcity of T-cell-specific data. Most studies on the HCMV TCR repertoire have focused on identifying specific or common TCR sequences, with few exploring their functional aspects or correlations with clinical practice. Therefore, future research on HCMV TCR sequences should address clinical challenges, assist risk stratification, and develop more accurate treatment regimens to guide clinical decision-making.

## Figures and Tables

**Table 1:** HLA-A02 and HLA-A24 restricted HCMV-associated TCR sequences.

Reported HLA Restriction	Reported Protein (Epitope Sequence)	Reported Sequence	Reported V Gene	Reported J Gene	Reference
A02	pp65 (MLNIPSINV)	CASSFAYGYTF	12.4	1.2	[87]
		CASSFGVNTEAFF	12.3	1.1	
		CASSFRGDTEAFF	12.4	1.1	
	pp65 (NLVPMVATV)	CAGSLVTGTGWGYTF	6.5	1.2	
		CASSFSTGTAGGYTF	6.5	1.2	
		CASSLDGVTGELFF	27	2.2	
		CASSLDRVTGELFF	27	2.2	
	pp65 (NLVPMVATV)	SSANYGY	8	1.2	[88]
		SSVNEA	8	1.1	
		SSVSGGASNEQ	13	2.1	
		SYATGTAYGY	13	1.2	
	IE1 (VLEETSVML)	CASSLDSIASGNTIYF	5–1	1.3	[89]
		CASSLQRGRTDTQYF	11–2	2.3	
		CASSLVSGGWTEAFF	11–2	1.1	
		CASSPDSQSSGNTIYTF	5–1	1.3	
	pp65 (NLVPMVATV)	CASNPMGQGILFF	9	2.2	
		CASSCQTGAACGYTF	6–5	1.2	
	pp65 (NLVPMVATV)	CASSLAPGATNEKLF	07-06*01	01-04*01	[78]
		CASASANYGYT	12	01-02*01	
A24	pp65 (QYPDVAALF)	CAGTGIRSAGELFF	30	2–2	[90]
		CASSLDTDTQYF	5	2–3	
		CASSLGAGGPSDTQYF	5	2–3	
		CASSLNSVGTEAFF	28	1–1	
		CASSSDNAIGGGSYGYTF	28	1–2	
		CASSSTGGGGAEAFF	7	1–1	
		CASSSTGGGGTEAFF	7	1–1	
		CASTPRDRSNYEQYF	27	2–7	
		CSARFSGQGTEAFF	20	1–1	

## Data Availability

No new data were created or analyzed in this study. Data sharing is not applicable to this article.
